# Death Receptor 3 regulates distinct pathological attributes of acute versus chronic murine allergic lung inflammation

**DOI:** 10.1016/j.cellimm.2017.09.005

**Published:** 2017-10

**Authors:** Ravinder Kaur Singh, William Victor Perks, Jason Peter Twohig, Emma J. Kidd, Kenneth Broadley, Stuart N. Farrow, Anwen Sian Williams, Philip Russel Taylor, Eddie Chung Yern Wang

**Affiliations:** a Division of Infection & Immunity, School of Medicine, Cardiff University, Heath Park, Cardiff CF14 4XN, UK; b School of Pharmacy and Pharmaceutical Sciences, Cardiff University, Cardiff CF10 3NB, UK; c CRT discoveries laboratories, Babraham Research Campus, Cambridge CB22 3AT, UK

## Abstract

The Death Receptor 3 (DR3)/Tumour Necrosis Factor-like cytokine 1A (TL1A) axis stimulates effector T cells and type 2 innate lymphocytes (ILC2) that trigger cytokine release and drive disease pathology in several inflammatory and autoimmune diseases, including murine models of acute allergic lung inflammation (ALI). The aim of this study was to elucidate the role of DR3 in *chronic* ALI compared to acute ALI, using mice genetically deficient in the DR3 gene (DR3^ko^). Results showed DR3 expression in the lungs of wild-type mice was up-regulated following induction of acute ALI and this increased expression was maintained in chronic disease. DR3^ko^ mice were resistant to cellular accumulation within the alveolar passages in acute, but not chronic ALI. However, DR3^ko^ mice displayed reduced immuno-histopathology and goblet cell hyperplasia; hallmarks of the asthmatic phenotype; in chronic, but not acute ALI. These data suggest DR3 is a potential therapeutic target, involved in temporally distinct aspects of ALI progression and pathogenesis.

## Highlights

• DR3 has distinctive roles in acute and chronic stages of allergic lung inflammation.
• In acute lung inflammation, DR3^ko^ mice are protected from lung cell infiltration.
• In chronic lung disease, DR3 is essential for goblet cell hyperplasia.
• Conclude that DR3 may be required for the development of lung pathology.

## 1 Introduction

Death Receptor 3 (DR3, TNFRSF25), along with its primary TNFSF ligand TL1A (TNFSF15), has recently emerged as a major regulator of inflammation and immunity, refereeing a range of cellular responses from differentiation and proliferation to cell death.

The effects of the DR3/TL1A pathway in disease are both varied and far-reaching. Loss of DR3 has been shown to impair both anti-bacterial [1] and anti-viral immunity [2], though conflicting data exists as to DR3’s impact during parasitic helminth infection [3], [4]. Most notably, DR3 has been shown to have a role in several autoimmune diseases, including rheumatoid arthritis (RA) [5], [6], [7], [8], [9], [10] and inflammatory bowel disease (IBD) [11], [12], [13], [14], [15], [16], [17], both of which are considered chronic in nature. Variation in the TNFRSF25 gene locus has been suggested as a risk factor for RA, with patients displaying elevated TL1A levels in serum and tissue [5], [8], [9], [18]. Furthermore, an *in vivo* antigen induced arthritis model showed mice genetically deficient in the DR3 gene (DR3^ko^) as presenting with reduced pathology and inflammatory cell infiltrate [6], [19]. This has been attributed to multiple DR3-driven functions which impact on effector T cell development, osteoclast differentiation [20] and cytokine/chemokine release. TNFSF15 has also been implicated in other bone disorders such as ankylosing spondylitis [21], [22] and IBD development [23]. TL1A levels were found to be up-regulated in Crohn’s disease patients, correlating with disease progression and severity [24], [25]. Those exhibiting high levels also suffered intestinal fibrostenosis and worsened inflammation in the small intestine, suggesting TL1A as a prognostic marker [17], [26]. *In vivo*, mice constitutively expressing TL1A developed colitis or ileitis [17], [27], [28].

Asthma, like RA, ankylosing spondylitis and IBD, is considered a chronic disease. Despite this, the majority of studies concerning DR3 in allergic lung inflammation have utilised acute disease models, sufficient to imitate early airway inflammatory events. DR3 was found to be central in the development of acute allergic lung inflammation, driving T cell accumulation [29] and IL9 production [30] in an OVA induced allergic model. Similarly, TL1A was found to co-stimulate type 2 innate lymphocyte (ILC2) expansion and promote IL13 production in a papain allergen model of acute lung inflammation [3], [4]. However asthma, due to its chronic nature, is typified by airway remodelling as well as airway inflammation [31], [32]. To discern the differential effects of DR3 in an acute versus a more physiologically relevant chronic model of allergic lung inflammation, DR3^ko^ mice were subjected to repeated allergen inhalation challenges, allowing both airway infiltrating cells and pathology to be examined. Our results show DR3 to be instrumental in chronic airway remodelling, promoting goblet cell hyperplasia and bronchiole pathology, implicating DR3 as a potential therapeutic target in asthmatic disease.

## 2 Methods

### 2.1 Animals

Age-matched female DR3^ko^ and DR3^wt^ littermates of 7–12 weeks of age were used in experiments. Mice were bred on a C57BL/6 background and derived from a DR3^het^ colony that was founded from animals provided by CRUK [33]. All procedures were approved by the Local Research Ethics Committee and performed in accordance with Home Office approved license PPL 30/2580.

### 2.2 OVA induced lung inflammation

As previously described [34], mice were sensitised on days 0 and 5 via an i.p. injection containing 100 µg chicken OVA (VWR) [34] emulsified with 50 mg aluminium hydroxide (Thermo Scientific). To study acute pulmonary inflammation, on day 15 mice were challenged twice, 4 h apart for 1 h via inhalation with a 0.5% (w/v) solution of OVA or PBS. Mice undergoing chronic allergic lung inflammation were exposed to nebulised 2% (w/v) OVA aerosol or PBS for 3 days per week for 6 weeks from day 15, resulting in a total of 18 inhalation challenges. Each challenge lasted 30 min, excluding the final one which lasted for 1 h. Inhalations were carried out in a Perspex box (Buxco Electronics) attached to a Wright nebuliser (Pulmostar, Devillbiss Healthcare) [34]. Cell infiltration was evaluated 24 h after the final challenge for both protocols in bronchoalveolar lavage fluid (BALF). Lungs were fixed in neutral buffered formalin for histological analysis.

### 2.3 Flow cytometry

Following the centrifugation of BAL fluid to isolate leukocytes, a total cell count was performed using a Neubauer Haemocytometer and trypan blue to exclude dead cells. Samples were then treated with anti-CD16/32 (BD Pharmingen) and stained at 4 °C for 25 min with the following pre conjugated mAbs against the indicated antigens: MHCII-FITC, NK1.1-FITC, CD4-PerCP Cy 5.5, CD11c-PECy7, B220-PE Texas Red, Ly6G-V450, CD44-V450, TCRβ-APC, CD11b-APCCy7, CD3-APC (all BD Biosciences); 7/4-PE (AbD Serotec), CD8-PECy7 (Invitrogen), F4/80-APC (Invitrogen). Samples were then washed before analysis on a CyAn™ ADP Flow cytometer using Summit software (Beckman Coulter). Cell subsets were defined as the following: eosinophils (F4/80^int^CD11b^int^SSC^hi^), 7/4^−^ monocytes (F4/80^low^CD11b^int^SSC^low^7/4^−^), 7/4^+^ monocytes (F4/80^low^CD11b^int^SSC^low^7/4^+^), myeloid dendritic cells (DCs) (CD11b^+^CD11c^+^MHCII^+^), CD4^+^ T cells (CD3^+^αβTCR^+^CD4^+^), CD8^+^ T cells (CD3^+^αβTCR^+^CD8^+^), NK (NK1.1^+^αβTCR^−^CD3^−^) and NKT (NK1.1^+^αβTCR^+^CD3^+^) cells.

### 2.4 Chemokine analysis

ELISAs were performed following manufacturers’ instructions using BALF supernatant. All ELISAs were obtained from R&D systems.

### 2.5 Histology

Lung tissue sections (5 μm) were stained with haematoxylin and eosin (H&E), Periodic acid-Schiff (PAS) or Van Gieson stain. H&E stained lungs were scored according to a 5 point mean pathology gradient; 0: normal lung, 1: minor perivascular inflammation and cell infiltrate, 2: slight perivascular and peribronchiolar inflammation, 3: moderate peribronchiolar and perivascular inflammation and airway cuffing, 4: marked peribronchiolar and perivascular inflammation and airway cuffing, 5: severe peribronchiolar inflammation, perivascular inflammation and airway cuffing (almost solid lung). PAS stained sections were quantified using Leica Qwin V3 software, with the % of goblet cells calculated as a % of the airway based upon positive staining. Van Gieson stained sections were judged according to the Ashcroft Score [35]; 0: normal lung, 1: minimal fibrosis thickening of alveolar/bronchial walls, 3: moderate thickening of walls without obvious damage to lung architecture, 5: increased fibrosis with definite damage to lung architecture and formation of fibrosis bonds/fibrosis masses, 7: severe distortion of architecture and large fibrosis area, 8: total fibrosis obliteration of field. All lung histology sections were blind scored by 2 individuals and scores averaged.

### 2.6 DR3 immunohistochemistry

Briefly, lung sections (5 μm) were rehydrated and endogenous peroxidise activity blocked. Following blocking, sections were stained with 20 μg/ml goat biotinylated anti-DR3 (R&D Systems) or isotype control for 2 h. Positive staining was visualised using a streptavidin HRP conjugate and DAB chromagen, before counterstaining with haematoxylin, dehydration and mounting in DPX. Images were captured using an Olympus Camedia C-3030 Digital Camera. Staining was simultaneous for sections and positive staining threshold was set using the isotype control. DR3 staining was quantified using Leica Qwin V3 software. Randomly selected areas were analysed for positive staining and calculated as a % of the total lung area shown.

### 2.7 Statistical analysis

All results are presented as the mean ± the standard error of the mean (SEM) and were analysed using GraphPad Prism v5. Unpaired *t* tests and two-way ANOVAs with Bonferroni post hoc test were used for analyses with more than 1 variable. *P* values of <0.05 were considered significant: ^∗^denotes *p* < 0.05, ^∗∗^denotes *p* < 0.01, ^∗∗∗^denotes *p* < 0.001.

## 3 Results

### 3.1 DR3 is significantly up-regulated in the lung in acute but not chronic allergic lung inflammation

**Fig. 1:**
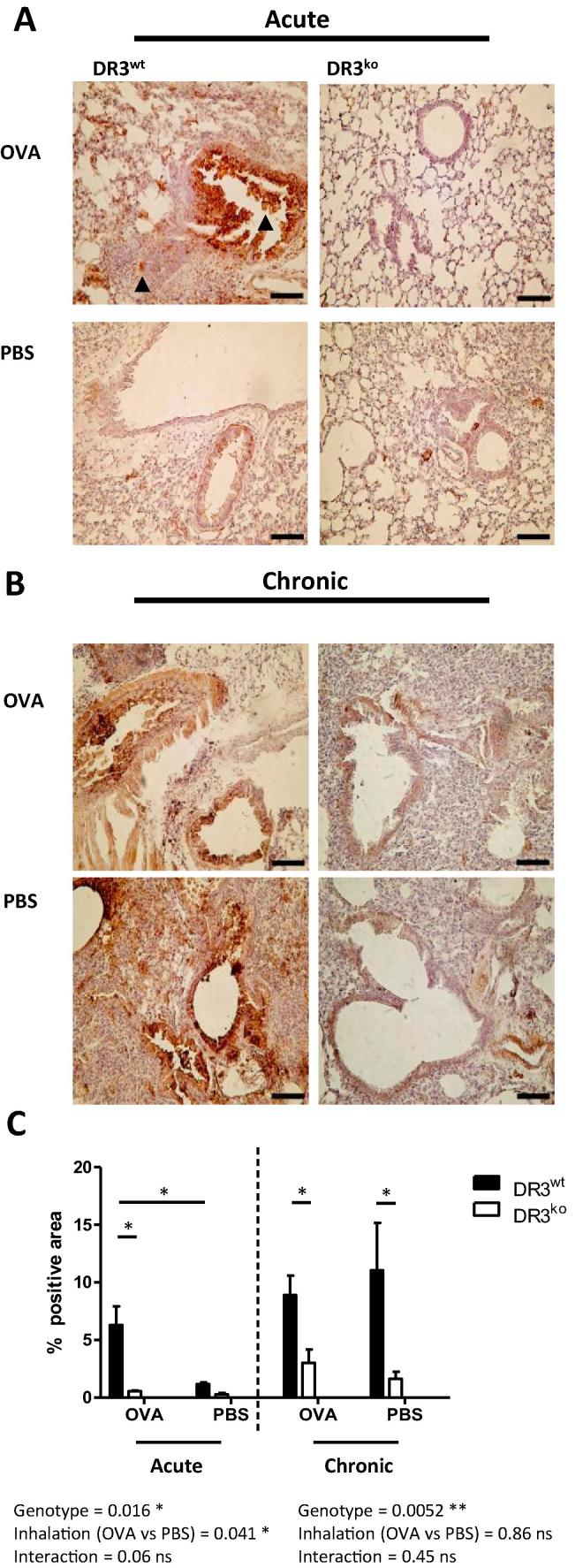
DR3 expression in the lung following acute and chronic allergic lung inflammation. Following OVA induced allergic lung inflammation, lungs were perfused, harvested, fixed, sectioned and stained for DR3 expression. Representative pictures showing DR3 staining in the lungs of DR3^wt^ and DR3^ko^ OVA treated mice along with DR3^wt^ and DR3^ko^ PBS challenged controls (A) 24 h after acute allergic lung inflammation and (B) 24 h after chronic allergic lung inflammation (scale bar = 100 µm). (C) Summary of DR3 expression in acute and chronically treated lungs. For acute lung inflammation, both genotype (*p* < *0.05*) and inhalation treatment (*p* < *0.05*) significantly effect DR3 expression. For chronic lung inflammation, genotype is a significant factor concerning DR3 expression (^**^p < *0.01*), whilst OVA/PBS inhalation treatment shows no significant effect. Mean % positive (brown) pixels within the lung shown ± SEM (*n* = 5 DR3^wt^ and DR3^ko^ mice per treatment). Significant differences were identified using two-way ANOVA with Bonferroni post-test comparing DR3 expression in acute and chronic experiments.

The DR3/TL1A pathway has been proved essential to the development of airway inflammation in an acute model of lung disease [4], [29], [36]. Nonetheless, little is known about the expression patterns of DR3 in the lung following OVA induced inflammation. To examine DR3 expression in the lung following both acute and chronic OVA induced inflammation, immuno-histochemical analysis was used. DR3 expression was found to be focused within the peribronchiolar areas of the lung, with occasional signal noted in the alveolar spaces (Fig.1a). DR3^wt^ acute OVA-treated lungs expressed 6.5 ± 3.0% DR3, significantly more than PBS-treated controls (1.2 ± 0.1%) (Fig.1c). Conversely, DR3^wt^ mice undergoing chronic OVA treatments displayed analogous DR3 levels to PBS controls but significantly more than DR3^ko^ mice, with expression again focused in the bronchiolar epithelium and occasionally the smooth muscle (Fig.1b). Therefore, whilst the locality of DR3 was similar in acute and chronically treated lungs, differences in the degree of expression were only observed in the acute model.

### 3.2 DR3 regulates cellular accumulation into the alveolar passage in acute but not chronic allergic lung inflammation

**Fig. 2:**
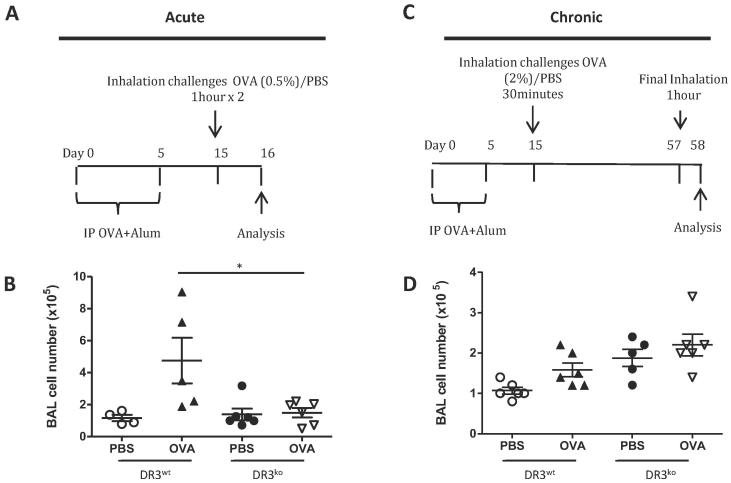
Total cell number in bronchoalveolar lavage fluid of DR3^wt^ and DR3^ko^ mice following acute and chronic allergic lung inflammation. Following OVA induced allergic lung inflammation, cells were isolated from the alveolar passage via lavage and counted. (A) Timeline of acute allergic lung inflammation sensitisation and challenge. (B) Total cell number in BALF 24 h after the final inhalation challenge in acute protocol. ^*^p < 0.05 using *t* test comparing DR3^wt^ and DR3^ko^ OVA challenged mice. (C) Timeline of chronic allergic lung inflammation sensitisation and challenge. (D) Total cell number in BALF 24 h after final inhalation challenge in chronic protocol.

**Fig. 3:**
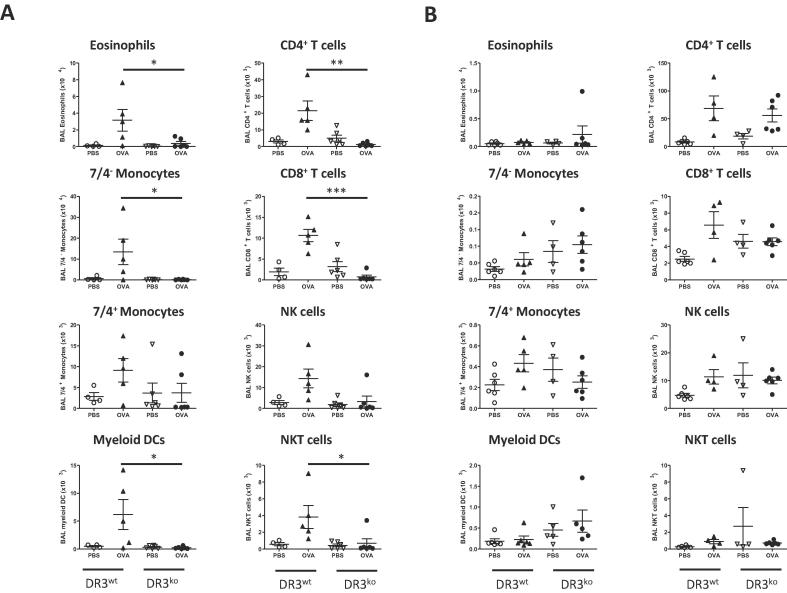
Leukocyte cell subset numbers in bronchoalveolar lavage fluid of DR3^wt^ and DR3^ko^ mice following acute and chronic allergic lung inflammation. Cell subsets numbers were calculated from BALF using cell counts and flow cytometry. (A) Individual cell subsets labelled eosinophils (^*^p < 0.05), 7/4^−^ monocytes (^*^p < 0.05), 7/4^+^ monocytes, myeloid DCs (^*^p < 0.05), CD4^+^ T cells (^**^p < 0.01), CD8^+^ T cells (^***^p < 0.001), NK cells and NK T cells (^*^p < 0.05). Significance tested using *t* test comparing DR3^wt^ and DR3^ko^ OVA challenged mice. (B) Individual cell subsets labelled eosinophils, 7/4^−^ monocytes, 7/4^+^ monocytes, myeloid DCs, CD4^+^ T cells, CD8^+^ T cells, NKT cells and NK cells. Values represent mean ± SEM. Each symbol represents data from a single mouse.

DR3^ko^ mice or mice treated with anti-TL1A blocking antibody, have been shown to be resistant to cellular accumulation in the alveolar passage following sensitisation and acute challenge with OVA. Our data is consistent with these results, as in our acute model, DR3^ko^ OVA treated mice had 1.5 ± 0.3 × 10^5^ cells within the BALF, significantly less than the 4.8 ± 1.4 × 10^5^ cells recorded in DR3^wt^ OVA treated mice and equivalent to the PBS controls in both DR3^wt^ and DR3^ko^ mice (Fig.2b). In contrast, in the chronic allergic lung inflammation model, no significant differences were found in BALF cell number between DR3^wt^ and DR3^ko^ groups, irrespective of whether they were challenged with OVA or control PBS (Fig.2d). Analysis of individual cell subsets following the induction of acute allergic lung inflammation revealed DR3^ko^ OVA mice had significantly fewer cells of both myeloid and lymphocytic origin, including eosinophils, 7/4^−^ monocytes, myeloid dendritic cells (DCs), CD4^+^, CD8^+^ and NKT cells. However, 7/4^+^ monocytes and NK cells, though diminished, were not significantly lower (Fig.3a). Despite the broad range of cell types affected, the decrease in DR3^ko^ cell numbers following acute OVA challenge was not due to defective chemokine production as seen in acute peritoneal inflammation [37], as no disparities were seen in CCL3, CCL4, CCL5, CXCL1, CXCL2, CXCL10 or CXCL13 levels in the BALF following the induction of lung inflammation (Table 1). In contrast, although chronic OVA challenged DR3^ko^ mice exhibited reduced CD4^+^ and CD8^+^ T cell numbers compared to DR3^wt^ mice, this decrease was not significant (Fig.3b). Thus, whilst DR3 is absolutely required for inflammatory cell infiltration following acute OVA challenge, it has a limited effect following chronic OVA aerosolisations, which in general showed little cellular accumulation regardless of the presence of DR3.

**Table 1:** Chemokine levels within the BAL of DR3^wt^ and DR3^ko^ mice following acute and chronic allergic lung inflammation. Following OVA-induced allergic lung inflammation, BAL fluid was isolated and the supernatant used to determine chemokine levels using ELISA. No significant differences were seen. Significance determined using *t* test comparing DR3^wt^ and DR3^ko^ OVA challenged groups. Values represents mean ± SEM (*n* = 6 DR3^wt^ and DR3^ko^ mice per treatment).

	Chemokine	DR3^wt^		DR3^ko^		Significance$
		PBS*	OVA^	PBS*	OVA^	
Acute	CCL3	N/A†	N/A†	N/A†	N/A†	N/A†
	CCL4	N/A†	N/A†	N/A†	N/A†	N/A†
	CCL5	74 ± 18	92 ± 10	77 ± 15	78 ± 18	*p* = 0.55 N.S.D
	CXCL1	N/A†	N/A†	N/A†	N/A†	N/A†
	CXCL2	90 ± 21	107 ± 7	144 ± 55	85 ± 18	*p* = 0.41 N.S.D
	CXCL10	310 ± 36	342 ± 26	359 ± 72	275 ± 33	*p* = 0.15 N.S.D
	CXCL13	229 ± 34	275 ± 51	456 ± 180	292 ± 39	*p* = 0.79 N.S.D

Chronic	CCL3	36 ± 10	31 ± 5	38 ± 4	27 ± 3	*p* = 0.46 N.S.D
	CCL4	N/A†	N/A†	N/A†	N/A†	N/A†
	CCL5	145 ± 40	125 ± 22	138 ± 21	96 ± 11	*p* = 0.26 N.S.D
	CXCL1	74 ± 16	45 ± 6	63 ± 5	48 ± 8	*p* = 0.79 N.S.D
	CXCL2	254 ± 54	216 ± 20	272 ± 18	185 ± 25	*p* = 0.35 N.S.D
	CXCL10	799 ± 144	955 ± 246	941 ± 90	666 ± 64	*p* = 0.28 N.S.D
	CXCL13	551 ± 99	556 ± 46	716 ± 32	483 ± 65	*p* = 0.37 N.S.D

N.S.D = no significant difference.

* PBS corresponds to mice challenged via inhalation with PBS.

^ OVA corresponds to mice challenged via inhalation with OVA.

† N/A corresponds to chemokines either not being present or below detection range.

$ Significance between DR3^wt^ and DR3^ko^ OVA treated groups tested using *t* test.

### 3.3 DR3 enhances airway pathology and goblet cell hyperplasia in chronic allergic lung inflammation

**Fig. 4:**
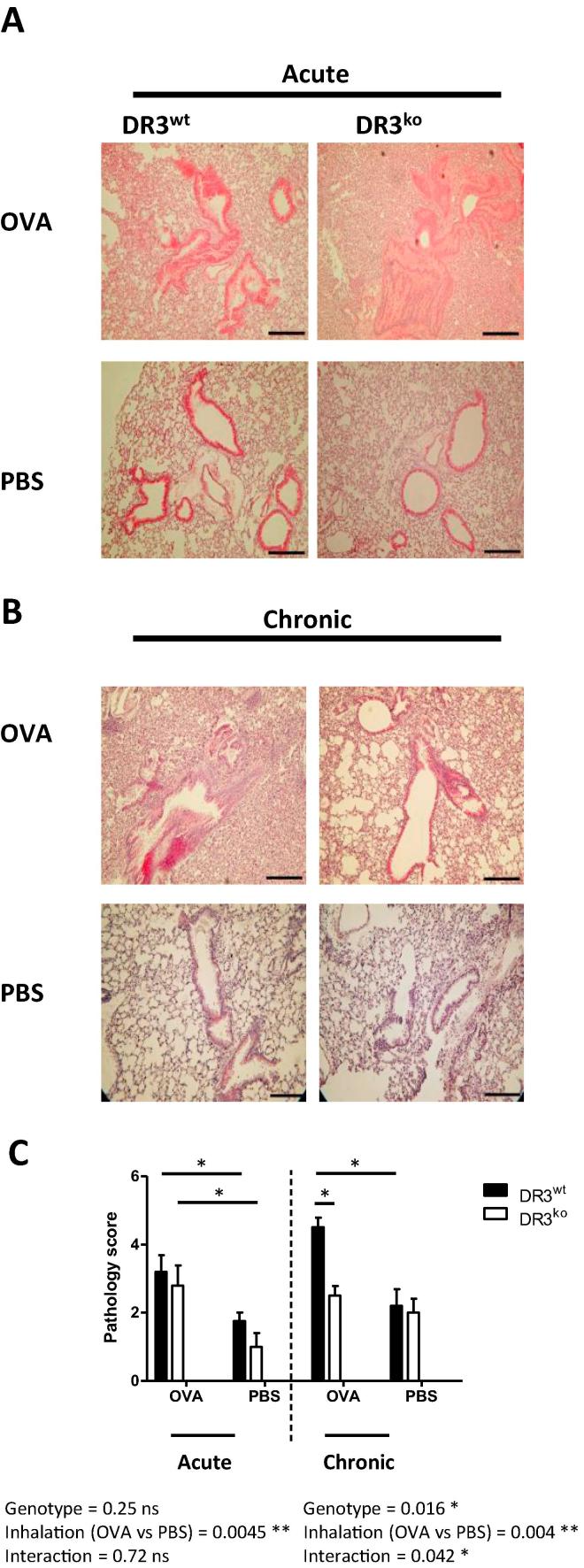
Lung pathology of DR3^wt^ and DR3^ko^ mice following acute and chronic allergic lung inflammation. Following OVA induced allergic lung inflammation, lungs were perfused, harvested, fixed, sectioned and stained with H&E for histological evaluation. (A) Representative pictures of H&E stained lungs (scale bar = 250 µm) following acute challenge with OVA or PBS (as labelled). (B) Representative pictures of H&E stained lungs (scale bar = 250 µm) following chronic OVA or PBS challenge (as labelled). (C) Summary of pathology scores for acute and chronic H&E stained lungs. For acute lung inflammation, inhalation treatment is significant for lung pathology (^**^p < *0.01*), whilst genotype is not. For chronic lung inflammation, both inhalation (^**^p < *0.01*) and genotype display significance, as DR3^ko^ OVA treated lungs showed significantly less inflammation than DR3^wt^ OVA challenged (^*^p < *0.05*). Lungs were blind scored by two individuals for pathology and an average taken for each lung. Results analysed using two-way ANOVA with Bonferroni post-test. Data represents mean ± SEM (*n* = 4–6 DR3^wt^ and DR3^ko^ mice per treatment).

**Fig. 5:**
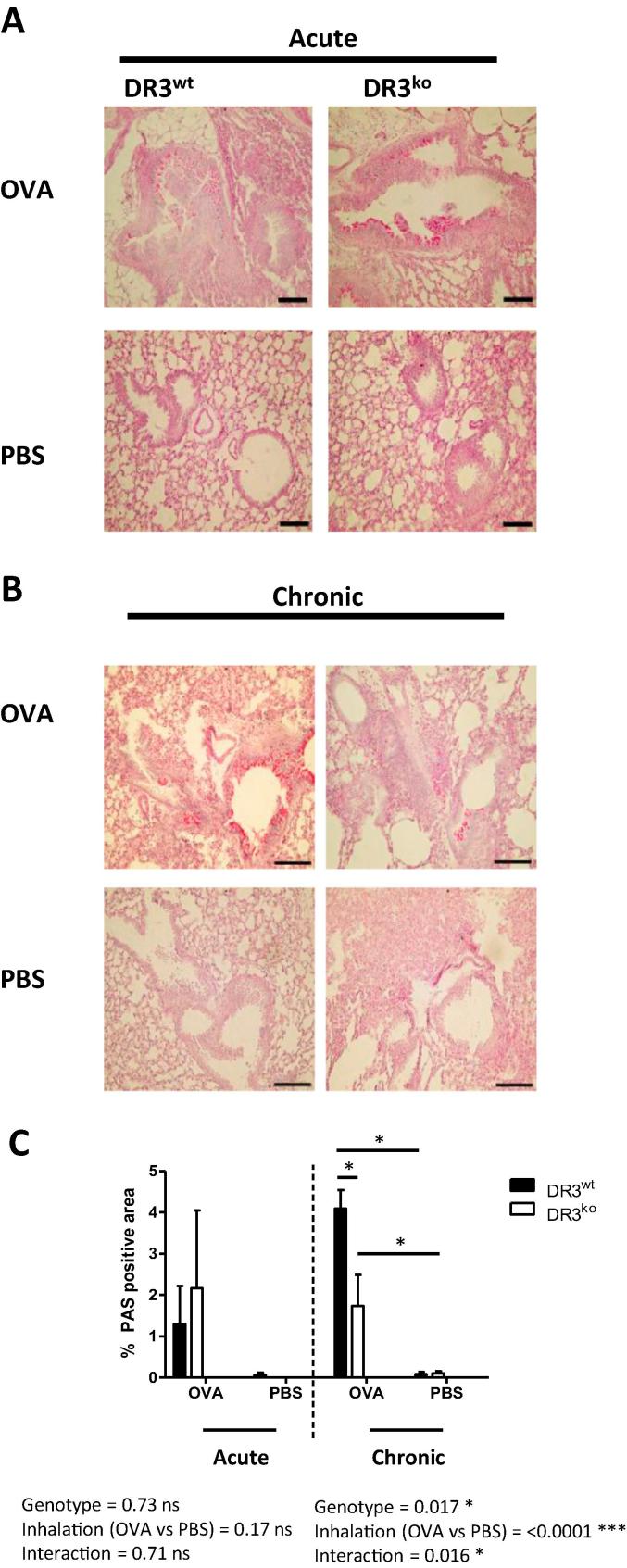
Goblet cell hyperplasia in the lungs of acute and chronically challenged DR3^wt^ and DR3^ko^ mice. Following OVA induced allergic lung inflammation, lungs were perfused, harvested, fixed, sectioned and stained with Periodic acid-Schiff (PAS) for histological evaluation. (A) Representative photos of PAS stained lungs (scale bar = 100 µm) following OVA or PBS control induced acute allergic lung inflammation (as labelled). (B) Representative pictures of PAS stained lungs (scale bar = 100 µm) following chronic OVA or PBS challenge. (C) Summary of goblet cell positive areas in acute and chronic allergic lung inflammation. Following acute inflammation, neither inhalation treatment nor genotype proved significant. Analysis of chronically challenged lungs showed DR3^wt^ OVA samples to have significantly more mucin producing goblet cells than DR3^ko^ OVA aerosolised mice, as genotype was a significant factor (^*^p *<* *0.05*). Inhalation treatment was also significant, as OVA treated mice had more goblet cells than PBS challenged animals (^***^p < 0.001). The area of PAS+ cells was taken as a % of the area surround and an average calculated for each lung. Results analysed using two-way ANOVA with Bonferroni post-test. Data represents mean ± SEM (*n* = 4–6 DR3^wt^ and DR3^ko^ mice per treatment).

**Fig. 6:**
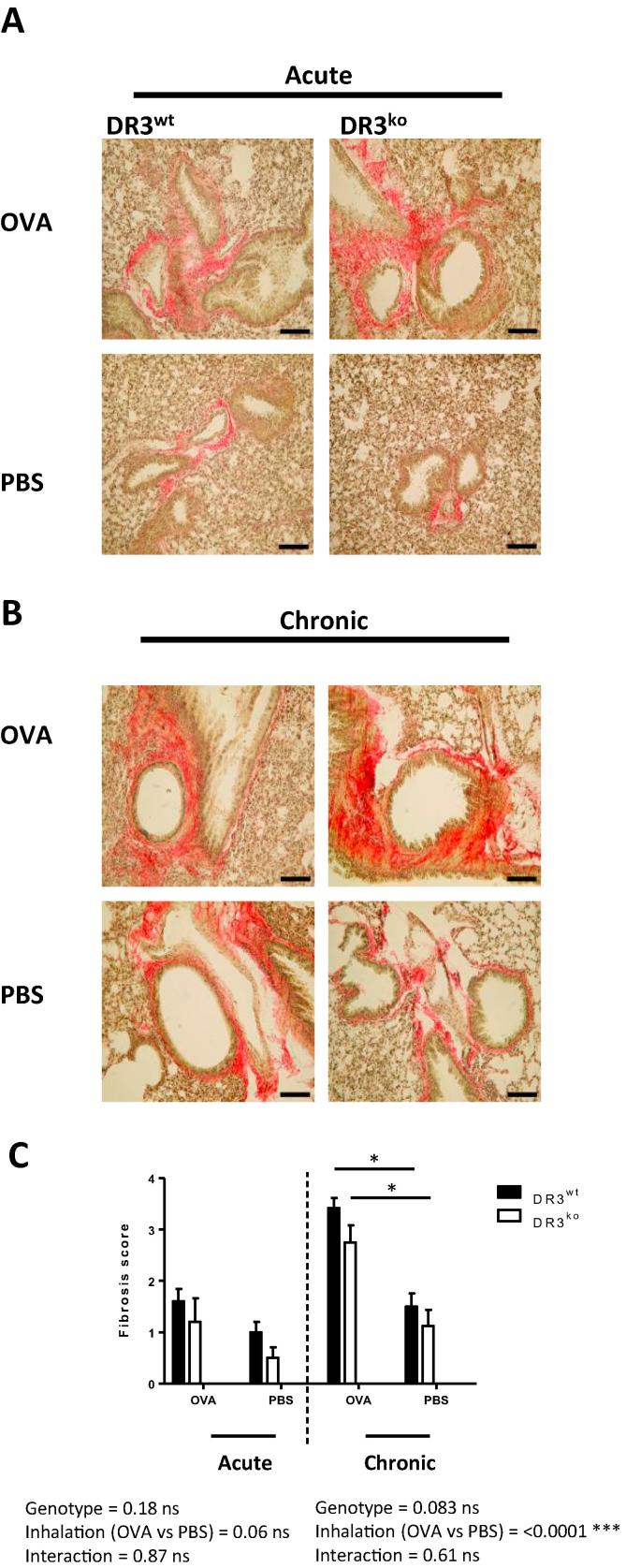
Fibrosis assessment of DR3^wt^ and DR3^ko^ lung following acute and chronic allergic lung inflammation. Following OVA induced allergic lung inflammation, lungs were perfused, harvested, fixed, sectioned and stained with Van Gieson solution for histological evaluation. (A) Representative photos of Van Gieson stained lungs (scale bar = 100 µm) post acute allergic lung inflammation (as labelled). (B) Representative pictures of Van Gieson stained lungs (scale bar = 100 µm) following chronic OVA or PBS challenge (as labelled). (C) Summary of fibrotic scores in acute and chronic lungs. Neither genotype nor inhalation treatment proved significant following acute allergic lung inflammation. Following chronic allergic lung inflammation, inhalation treatment was shown to be significant to fibrosis development as OVA challenged mice displayed significantly more fibrosis than PBS treated lung (^***^p < *0.001*). Genotype was not a significant factor. Lungs were blind scored by two individuals using Ashcroft score of fibrosis and an average taken for each lung. Results analysed using two-way ANOVA with Bonferroni post-test. Data represents mean ± SEM (*n* = 4–6 DR3^wt^ and DR3^ko^ mice per treatment).

To determine the pathological consequences of DR3 signalling in the lung following OVA acute and chronic challenge, we used histological analysis to assess general pathology, including goblet cell hyperplasia and fibrosis, both of which represent airway remodelling. This complex and dynamic process is thought to contribute to the dysregulation of airway function, therefore prolonging the allergic response and typifying human asthma. H&E staining was used to assess general lung pathology. Staining revealed that although DR3 had no role in acute airway inflammation pathology (Fig.4a), in chronic allergic lung inflammation, DR3^ko^OVA challenged mice exhibited less lung inflammation compared to DR3^wt^ OVA mice; 2.5 ± 0.3 vs 4.5 ± 0.3, respectively (Fig.4b and c). Both inhalation treatment and genotype were deemed significant by two-way ANOVA, as was the interaction between the two variables. This was highlighted by decreased peribronchial inflammation and cellular cuffing of the DR3^ko^ OVA challenged airways (Fig.4b). To quantitate levels of mucin producing goblet cells, lungs were stained with Periodic acid-Schiff (PAS) and evaluated using software to identify positive (pink) areas. Analysis of goblet cells following acute allergic lung inflammation indicated no significant differences between DR3^wt^ and DR3^ko^ OVA treated lungs (Fig.5a). However, DR3^ko^ lungs subjected to chronic OVA challenge had significantly less mucin-producing cells than their DR3^wt^ counterparts (Fig.5b and c), as again a significant interaction was noted between inhalation treatment and genotype. Lungs were also stained with Van Gieson solution to assay the level of collagen and thereby acquire an arbitrary measure of fibrosis. These levels were scaled against the Ashcroft score of fibrosis [35]. There was no significant differences between the lungs of OVA sensitised and challenged DR3^ko^ mice compared to DR3^wt^ mice in either the acute or chronic models of lung inflammation (Fig. 6). Unsurprisingly however, mice subjected to multiple OVA inhalations presented with higher fibrotic scores than mice that had undergone the acute protocol, regardless of genotype. Our data shows that absence of DR3 protects against the development of certain aspects of severe immunopathology in repeated OVA induced chronic lung inflammation. Whilst fibrosis was not different, goblet cell hyperplasia and cellular infiltration in the perivascular, interstitial and peribronchial regions were substantially reduced in chronic DR3^ko^ OVA treated mice, suggesting DR3 as important for the development of some, but not all, pathological aspects in the airway remodelling process.

## 4 Discussion

The importance of DR3/TL1A in animal models of acute allergic lung disease are well established, despite asthma predominantly being considered a chronic syndrome. The results of this study indicate that whilst DR3 induces a multitude of effects upon the induction of allergic lung inflammation, these are highly dependent upon the model employed. In acute disease, DR3 was found to be responsible for cellular accumulation in the alveolar passage. This was concurrent with DR3 up-regulation in the lung. Interestingly however, during the chronic phase of disease, DR3 promoted airway remodelling via goblet cell hyperplasia and immuno-pathology, including bronchiole and parenchyma cell infiltration.

Models of acute allergic lung inflammation have long been employed to identify the mechanisms underlying the immunological and inflammatory responses of asthma, replicating the initiating events leading to disease. Multiple studies have previously identified DR3 as a regulator of BALF cell accumulation, citing ILC2 [3], [4], Th2 [29], [36] and Th9 cells [30] as perpetrators. Data shown here concurs with published results, as DR3^ko^ allergen challenged mice exhibited reduced numbers of multiple cell types including eosinophils and CD4^+^ T cells. Furthermore, increased DR3 expression observed in DR3^wt^ OVA challenged lungs may be linked to the increased numbers of leukocytes in BALF, many of which are known to express DR3 [2], [36]. Multiple human studies have reported up-regulation of DR3 in tissues in inflammatory conditions, including psoriatic skin lesions [38] and renal tubular epithelial cells during acute transplant rejection [39]. Furthermore, recent human studies show DR3 to be highly expressed in lung biopsies obtained from active sarcoidosis patients [40], whilst in a murine model, TL1A has been shown to be expressed following papain induced lung inflammation [30]. However, this is the first report of DR3 expression in the murine lung, and more importantly, its increased expression in response to inflammation. Despite the differences noted in DR3 expression following acute ALI, equivalent degrees of lung immuno-pathology were noted between DR3^wt^ and DR3^ko^ OVA acute challenged mice. This is in contrast to previous published data, which described reduced histopathology scores, including peribronchial inflammation in DR3^ko^ mice [29] whilst dominant negative DR3 lungs exhibited reduced numbers of infiltrating cells around blood vessels and bronchiole [36]. There are several possible reasons for this discrepancy, the most likely being the use of different sensitisation and challenge protocols, which have previously been suggested as a reason for conflicting results within the literature of murine asthma models [41]. *Meylan* et al. sensitised mice with an OVA/Alum mix on days 0 and 7, followed by an intra-tracheal challenge on day 14 and intranasal challenge on day 15 [29] whereas here, mice were sensitised with an OVA/Alum emulsion on days 0 and 5, followed by two OVA aerosolisation challenges on day 15. Comparing these protocols, possibly the largest immunological impact may come from the route of final antigenic challenge. Intratracheal/intranasal challenge would lead to a larger antigenic dose in the upper airways, whilst aerosolisation would supply smaller doses diffusely to the lower airways [42], [43]. Overall, it appears that whilst short-term exposure is sufficient to imitate airway inflammatory events [44], the model of acute allergic lung inflammation employed herein does not exhibit the characteristics of airway remodelling, which generally occur as a result of chronic exposure.

Asthma is defined as a disease of the airways, characterised by airway inflammation, airway hyper-responsiveness and airway remodelling [45]. Although no single *in vivo* model is able to replicate all of these morphological and functional features, animal models have been used to study specific aspects of the disease. In this model of chronic allergic lung inflammation, it was found that airway inflammation, studied via cell accumulation, and allergic antibody production (data not shown) were not DR3 dependent. This is further supported by the lack of differences noted in chemokine concentrations, which were previously identified as DR3 dependent during acute peritoneal fibrosis [37] but not after viral challenge [2]. Moreover, none of the BALF cell subsets studied differed significantly between DR3^wt^ and DR3^ko^ mice following multiple OVA aerosolisation challenges. This is a common occurrence when modelling chronic allergic lung protocols, which continue to be problematic in exhibiting all the characteristics of human asthma [46]. *Koerner-Rettberg* et al. noted sustained airway lymphocytosis but short lived eosinophilia upon long term challenge [47]. Meanwhile, *Swirski* et al. describes diminishing eosinophilia by 3 weeks and complete resolution after 4 weeks of antigen exposure [48], suggesting that chronic exposure to antigen does not sustain airway inflammation but rather leads to airway unresponsiveness [49]. This apparent lack of contribution to airway inflammation after multiple aerosol challenges is not just restricted to DR3 amongst the TNFRSF. TNFR^ko^ mice also showed no differences compared to WT mice in BAL leukocyte numbers after 7 days of consecutive OVA aerosolisation, suggesting that removal of TNFα signalling alone is unable to abrogate allergic lung inflammation and other cytokines have the ability to compensate for its loss [50]. Furthermore, though not studied here, regulatory T cells (T_reg_) may also have contributed to the lack of airway inflammation seen. Functional effects of DR3 signalling on the T_reg_ subset have been studied, as both an agonistic DR3 antibody [51] and TL1A-IgG fusion protein [52] were reported to expand the pool of T_regs_ within the lung, reversing the ratio of conventional T cells to T_regs_. This was reported to lead to the suppression of acute allergic lung inflammation [51], a phenomenon which could also be active in the chronic stages of disease. Having said this, mice constitutively expressing TL1A on DCs displayed increased T_reg_ turnover, with pro-inflammatory signals over-riding suppressive effects [28], suggesting the relationship between DR3/TL1A and T_regs_ as both complex and not well defined.

Whilst chronic models of allergic lung disease have been shown to have modest levels of infiltration, airway remodelling is considered a fundamental feature of the disease. Intriguingly, despite flow cytometry results suggesting few differences in BALF leukocyte accumulation, H&E stained lung tissue showed greater cellular infiltration in the peribronchial and perivascular areas of DR3^wt^ OVA challenged mice, implying DR3 is involved in the regulation of lung pathology. There may be several reasons why BALF cell infiltration and pathology scores do not correlate, the first being due to the compartmentalised nature of the lung [53]. The lavage of the bronchoalveolar space is unlikely to represent the whole lung, hence the importance of whole lung pathology, as assessed here by H&E staining. Secondly, only immune cells were analysed in the BALF fluid obtained following the induction of allergic lung inflammation. The increased infiltration observed by histology could be due to an increase in structural cells such as fibroblasts, which have been shown to contribute to inflammatory events within the lung [54], [55]. However, without more immuno-histochemical staining using Abs that specifically identify such cells, this is difficult to assess.

As well as general lung pathology, DR3^ko^ OVA challenged mice also had lower levels of mucin producing goblet cells than DR3^wt^ OVA mice following chronic allergic lung inflammation. Mucin production is a key contributor to asthma and via the production of mucus plugs, also to fatalities [49], [56]. DR3 has already been shown to have a role in goblet cell hyperplasia, albeit in a model of small intestinal inflammation [26], [27], [28]. Mice which constitutively expressed TL1A on both T cells [27] and DCs [28] spontaneously developed small bowel disease which was characterised by goblet cell hypertrophy and hyperplasia, thought to be IL-13 driven. More recently, *Moltke* et al. suggest small intestine epithelial tuft cell derived signals to be the key activators of ILC2s and thereby IL-13 and goblet cell hyperplasia [57], suggesting perhaps TL1A could be released by tuft cells and activate ILC2s in this manner.

When studying the fibrotic response, there were no significant differences in collagen production between DR3^wt^ and DR3^ko^ OVA mice. The DR3/TL1A pathway has previously been shown to be involved in both intestinal [13], [17], [26], [58] and peritoneal fibrosis [37]. Mice which constitutively expressed TL1A exhibited enhanced gut fibrosis compared to WT mice [13], which correlated with an increase in TGFβ1 [26], a known mediator of fibrosis [59]. Similarly in the peritoneum, DR3^wt^ mice which received multiple inflammatory challenges displayed thickening of the peritoneal mesothelial layer and increased deposition of collagen [37]. Interestingly, other TNFR members have been found to have a role in the fibrotic response during chronic allergic lung inflammation. Inhibition of LIGHT led to a reduction in the level of fibrosis as well as smooth muscle mass, thought to be mediated via the inhibition of TGFβ and IL-13 release [60]. Similarly, TNFR p55/p75 deficient mice demonstrated significantly reduced peribronchial fibrosis, smooth muscle layer and deposition of extracellular matrix proteins in a model of chronic allergic lung inflammation [61]. The lack of differences shown here suggests that amongst the TNFRSF, DR3 is not essential for the initiation and development of lung fibrosis.

The relationship between airway inflammation and airway remodelling is poorly understood. This is made more difficult by the variety of sensitisation and challenge protocols used as well as the array of read-outs used to assess the responses. Data here corroborates published literature stating that loss of DR3 function ameliorates acute allergic lung inflammation, shown by reduced cellular infiltration into the BALF. In a more clinically relevant model of chronic allergic lung inflammation, DR3^ko^ OVA mice exhibited lower mucin levels and inflammation in the lung parenchyma compared to DR3^wt^ mice, although the underlying mechanisms behind this are unknown. This is the first report of DR3 in chronic allergic lung inflammation and its potential as a therapeutic target for antagonism of goblet cell hyperplasia and associated mucus over-production as a result of allergic disease.
